# The short third intracellular loop and cytoplasmic tail of bitter taste receptors provide functionally relevant GRK phosphorylation sites in TAS2R14

**DOI:** 10.1074/jbc.RA120.016056

**Published:** 2021-01-16

**Authors:** Donghwa Kim, Maria Castaño, Lauren K. Lujan, Jung A. Woo, Stephen B. Liggett

**Affiliations:** 1 Department of Medicine, University of South Florida Morsani College of Medicine, Tampa, Florida, USA; 2 Department of Molecular Pharmacology and Physiology, University of South Florida Morsani College of Medicine, Tampa, Florida, USA; 3 Departments of Medicine and Molecular Pharmacology and Physiology, University of South Florida Morsani College of Medicine, Tampa, Florida, USA

**Keywords:** TAS2R, desensitization, G protein–coupled receptors, G protein–coupled receptor kinases, β-arrestin, AR, adrenergic receptor, [Ca^2+^]_i_, intracellular calcium, CT, carboxy-terminal tail, DPD, diphenhydramine, EEA1, early endosome antigen 1, ERK1/2, extracellular signal–regulated kinase 1/2, GPCR, G protein–coupled receptor, GRK, G protein–coupled receptor kinase, GST, glutathione-*S*-transferase, HASM, human airway smooth muscle, IL3, third intracellular loop, PLA, proximity ligation assay, TAS2R, bitter taste 2 receptor

## Abstract

For most G protein–coupled receptors, the third intracellular loop (IL3) and carboxy-terminal tail (CT) are sites for G protein–coupled receptor kinase (GRK)–mediated phosphorylation, leading to β-arrestin binding and agonist-specific desensitization. These regions of bitter taste receptors (TAS2Rs) are extremely short compared with the superfamily, and their function in desensitization is unknown. TAS2R14 expressed on human airway smooth muscle cells relax the cell, suggesting a novel target for bronchodilators. To assess IL3 and CT in agonist-promoted TAS2R14 desensitization (tachyphylaxis), we generated fusion proteins of both the WT sequence and Ala substituted for Ser/Thr in the IL3 and CT sequences. *In vitro*, activated GRK2 phosphorylated WT IL3 and WT CT proteins but not Ala-substituted forms. TAS2R14s with mutations in IL3 (IL-5A), CT (CT-5A), and in both regions (IL/CT-10A) were expressed in human embryonic kidney 293T cells. IL/CT-10A and CT-5A failed to undergo desensitization of the intracellular calcium response compared with WT, indicating that functional desensitization by GRK phosphorylation is at residues in the CT. Desensitization of TAS2R14 was blocked by GRK2 knockdown in human airway smooth muscle cells. Receptor:β-arrestin binding was absent in IL/CT-10A and CT-5A and reduced in IL-5A, indicating a role for IL3 phosphorylation in the β-arrestin interaction for this function. Agonist-promoted internalization of IL-5A and CT-5A receptors was impaired, and they failed to colocalize with early endosomes. Thus, agonist-promoted functional desensitization of TAS2R14 occurs by GRK phosphorylation of CT residues and β-arrestin binding. However, β-arrestin function in the internalization and trafficking of the receptor also requires GRK phosphorylation of IL3 residues.

G protein–coupled receptors (GPCRs) are now recognized as multifunctional signaling units, with the capacity to evoke intracellular events mediated by G protein and non-G protein mechanisms (1). These events include mechanisms that act to coarse- and fine-tune their signaling over short- and long-term periods, such that cells expressing hundreds of receptors can achieve integrative physiologic actions in response to changing needs (2). The attenuation of receptor responsiveness (termed desensitization) may be particularly germane when therapeutic agonists are targeted to GPCRs, since this may lead to a loss of effectiveness (tachyphylaxis). The mechanism of agonist-promoted desensitization for many GPCRs is by phosphorylation by G protein–coupled receptor kinases (GRKs) of intracellular residues, typically in the third intracellular loop (IL3) or the cytoplasmic carboxy-terminal tail (CT) (3, 4). Some receptors also are phosphorylated at intracellular residues by kinases, such as PKA and PKC, which act to decrease receptor function (5, 6). Since this form of regulation can be evoked by heterologous means, it is not considered to be strictly agonist dependent (homologous). The GRK-phosphorylated receptor landscape (7), evoked by conformational changes from agonist binding, acts as a substrate for the binding of the nonvisual arrestins (β-arrestins) (8) at their N-terminal domains (9). The recruited β-arrestin subsequently binds *via* its fingerloop region to a cytoplasmic cavity of the receptor, sterically interrupting binding of the α-subunit of the G protein (10, 11, 12). This competition between the β-arrestin and G protein is often termed “uncoupling” because it acts to attenuate the G protein–mediated signal, such as cAMP production from Gs-coupled receptors or intracellular calcium [Ca^2+^]_i_ release from Gi- and Gq-coupled receptors. Recruited β-arrestins also bind clathrin and other cellular components that lead to receptor internalization to the cell interior *via* clathrin-coated pit endocytosis (13). This internalization may serve to further limit cellular responsiveness and also acts to initiate receptor degradation over prolonged agonist exposure. Paradoxically, these attenuation functions are also accompanied by an activated state of β-arrestin, which leads to *de novo* signaling that is independent of G protein coupling (1). GRK-mediated phosphorylation appears to be highly sensitive to the conformation of the intracellular loop or tail of GPCRs, and indeed, there is no identified “consensus” sequence for GRK phosphorylation and β-arrestin binding. In fact, some GPCRs with multiple potential phosphorylation sites fail to undergo GRK-mediated phosphorylation and agonist-dependent desensitization (14, 15, 16). Thus, the prediction of GRK-mediated events is not possible based on primary amino acid sequence of a given receptor. Furthermore, it appears that the interactions of bound arrestins within the receptor core display structural plasticity and are capable of adopting unique conformations depending on the receptor (11, 17, 18). Thus, for each GPCR of interest, specific studies are necessary to establish the sites and roles of GRK-mediated phosphorylation and β-arrestin engagement in cellular signaling.

In the current work, we have undertaken such studies with the bitter taste 2 receptor member 14 (TAS2R14), a widely expressed receptor that may play a role in metabolic and inflammatory diseases, and has been considered a target for a new class of bronchodilators because of its expression on human airway smooth muscle (HASM) cells. In these cells, TAS2R14 agonists stimulate [Ca^2+^]_i,_ causing hyperpolarization of the HASM cell membrane and marked HASM relaxation (19). This has brought forth the concept that TAS2R14 agonists might be useful for treating obstructive lung disease where HASM contraction plays an active role in airway obstruction. Understanding mechanisms that may be in play for agonist-mediated regulation of TAS2R14 is thus a necessary component for such a consideration.

## Results

### GRK2 phosphorylates intracellular TAS2R14 peptides at Ser and Thr residues

**Figure 1:**
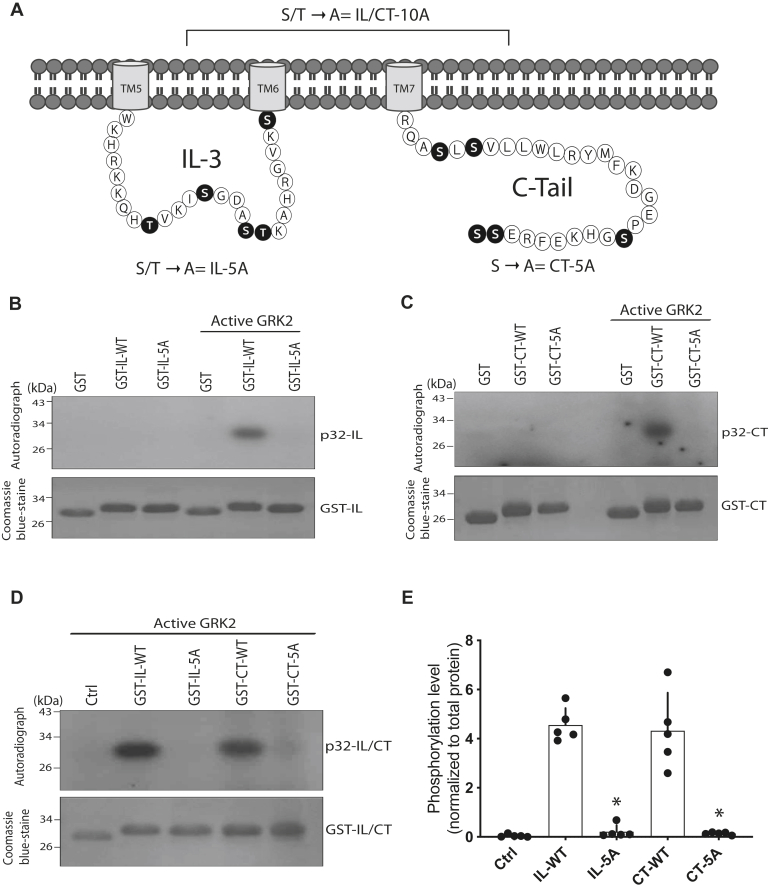
**TAS2R14-based peptides are phosphorylated at Ser/Thr by GRK2.***A*, localization of the Ser/Thr in the third intracellular loop (IL3) and C-terminal tail (CT) of TAS2R14. *B*–*D*, peptides based on WT IL3 and CT, but not Ala substituted for Ser/Thr peptides, are phosphorylated by activated GRK2 (representative experiments). *E*, GST-IL-WT and GSTCT-WT are phosphorylated by activated GRK2 to the same extent. The phosphorylation levels were normalized to the amount of loaded protein as determined by Coomassie blue staining. The small amount of signal observed in the mutant peptide lanes was not different than “0” as determined by the one-sample *t* test. ∗*p* < 0.001 *versus* analogous WT peptide. Data are shown as mean ± SD, with individual results, from five independent experiments. GRK2, G protein–coupled receptor kinase 2; GST glutathione-*S*-transferase; TAS2R14, bitter taste 2 receptor member 14.

The TAS2R14 has five Ser/Thr in the IL3 and five Ser in the cytoplasmic CT. These sequences were screened for potential GRK phosphorylation sites by expressing the glutathione-*S*-transferase (GST)–tagged fusion proteins representing WT TAS2R14 sequence or the analogous sequence with Ser/Thr substituted with Ala. IL3 peptides are denoted GST-IL-WT and GST-IL-5A for WT and mutant, respectively. The CT peptides are denoted GST-CT-WT and GST-CT-5A for the WT and mutant, respectively. *In vitro* phosphorylation experiments were carried out with the four proteins and active GRK2 or PKB (which acted as a negative control). None of the GST-tagged fusion proteins underwent phosphorylation in the absence of GRK2 (Fig. 1, *B* and *C*). In the presence of active GRK2, GST-IL-WT and GST-CT-WT were phosphorylated (Fig. 1, *B* and *C*), whereas the Ala-substituted proteins GST-IL-5A and GST-CT-5A were not (Fig. 1, *B* and *C*). In experiments where the proteins were electrophoresed on the same gel with equal protein loading, we found that the third loop peptide and the C-tail peptide phosphorylated to the same extent by active GRK2 (Fig. 1, *D* and *E*). These peptides do not have a consensus sequence for PKB, and indeed none were phosphorylated by PKB (Fig. S1).

### Functional consequences of TAS2R14 with mutated GRK2 phosphoacceptor sites

**Figure 2:**
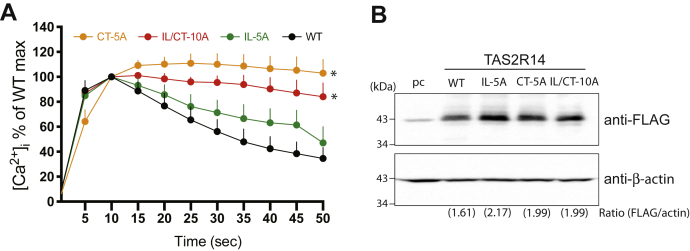
**Functional agonist-promoted desensitization profiles of the [Ca**^**2+**^**]**_**i**_**response of WT and mutant TAS2R14 receptors.***A*, results (mean ± SE) from five experiments. ∗Slope from 10 to 50 s as determined by linear regression differs from WT, *p* < 0.01 (see also Table 1). *B*, representative Western blot showing that expression of the WT and mutant receptors was comparable for experiments shown in panel *A*. [Ca^2+^]_i_, intracellular calcium; CT, carboxy-terminal tail; IL, intracellular loop; TAS2R14, bitter taste 2 receptor member 14.

To ascertain the functional effects of potential GRK phosphorylation of TAS2R14, three mutant receptor complementary DNAs were constructed substituting Ala in the encoded receptor for all Ser/Thr in the IL3 (IL-5A), the cytoplasmic tail (CT-5A), or both (IL/CT-10A). WT and mutant receptors were expressed in HEK-293 cells, and [Ca^2+^]_i_ release quantitated in real time for 45 s after exposure of the cells to the TAS2R14 agonist diphenhydramine (DPD). Nontransfected cells had no detectable [Ca^2+^]_i_ response (Fig. S2). Representative results from the TAS2R14-transfected cells are shown in Figure S2, and mean results from multiple experiments are shown in Figure 2*A*. WT-expressing cells showed a rapid increase in [Ca^2+^]_i_ that peaked after ∼10 s and then decreased (Fig. S2), with a mean slope of the decline from 10 s to 50 s being −1.51 ± 0.194 (Fig. 2*A* and Table 1). In contrast, IL/CT-10A, which lacks all 10 potential phosphoacceptor sites in the third loop and the CT, exhibited very little decrease in signaling, with a slope that was approximately sevenfold lower in absolute value than WT (*p* < 0.003: Fig. 2*A* and Table 1). In a similar manner, the CT mutant CT-5A failed to desensitize. Indeed, the slopes of the [Ca^2+^]_i_ responses of IL/CT-10A and CT-5A were not statistically different (*p* = 0.14; Fig. 2*A*). On the other hand, the third loop mutant IL-5A displayed [Ca^2+^]_i_ responses that were similar to R14-WT, with no statistical difference in the slopes (*p* = 0.127; Fig. 2*A* and Table 1). Mean expression levels of each of the receptors were comparable (Fig. 2*B* shows a representative Western blot). Taken together, these results suggested that despite both regions displaying GRK-mediated phosphorylation *in vitro*, functional desensitization of TAS2R14 requires only phosphorylation of one or more of the C-terminal Ser residues.

**Table 1:** Desensitization of TAS2R14

Plasmid/siRNA	Slope (N = 5)	*p Versus* WT/siCtrl
HEK-293T cells		
WT	−1.51 ± 0.19	REF
IL/CT-10A	−0.22 ± 0.23	<0.003
CT-5A	0.44 ± 0.34	<0.002
IL-5A	−0.99 ± 0.23	0.127
HASM cells		
SiCtrl	−1.74 ± 0.03	REF
siGRK2	−0.03 ± 0.35	<0.001

Transfected HEK-293T cells (*upper section*) expressing the indicated receptors were exposed to agonist and [Ca^2+^]_i_ monitored in real time. In the *lower section*, the same experiments were performed using HASM cells, which were transfected with siRNA constructs targeting GRK2 (siGRK2) or scrambled control (siCtrl). The slopes of the responses from 10 to 50 s were determined by linear regression and compared with that of WT in the case of the HEK-293 experiments or siCtrl in the case of HASM cells. REF is the reference for statistical comparison.

**Figure 3:**
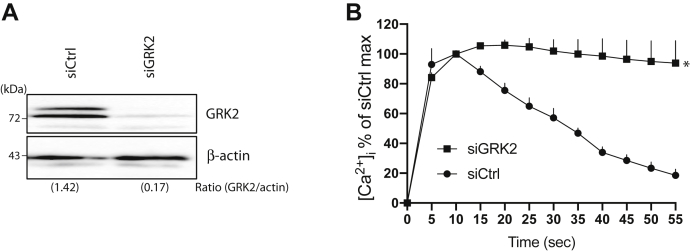
**Agonist-promoted desensitization of the TAS2R14 [Ca**^**2+**^**]**_**i**_**response is attenuated by GRK2 knockdown.***A*, representative Western blot showing the extent of GRK2 knockdown in cells used for experiments in panel *B*. *B*, results (mean ± SE) from five experiments. ∗Slope from 10 to 50 s as determined by linear regression is different from control (scrambled) siRNA, *p* < 0.01 (see also Table 1). [Ca^2+^]_i_, intracellular calcium; GRK, G protein–coupled receptor kinase; TAS2R14, bitter taste 2 receptor member 14.

To further implicate GRK phosphorylation as a mechanism of rapid desensitization, and, to ascertain relevance in a cell type of interest, cultured HASM cells were utilized to ascertain [Ca^2+^]_i_ responsiveness in siCtrl- and siGRK2-transfected cells (Fig. S3). These cells have been previously shown to endogenously express TAS2R14 and GRK2 (19, 20). In the siGRK2-transfected cells, GRK expression was decreased by >85% compared with siCtrl-transfected cells (Fig. 3*A*). The [Ca^2+^]_i_ response to agonist (Fig. 3*B*) was markedly altered by the GRK2 knockdown, revealing a pattern consistent with less desensitization, with mean slope of −0.03 ± 0.35 *versus* −1.74 ± 0.03 for siCtrl-transfected cells (*p* < 0.001).

### β-arrestin2 recruitment and binding to TAS2R14 is dependent on IL3 and C-terminal GRK phosphorylation sites

**Figure 4:**
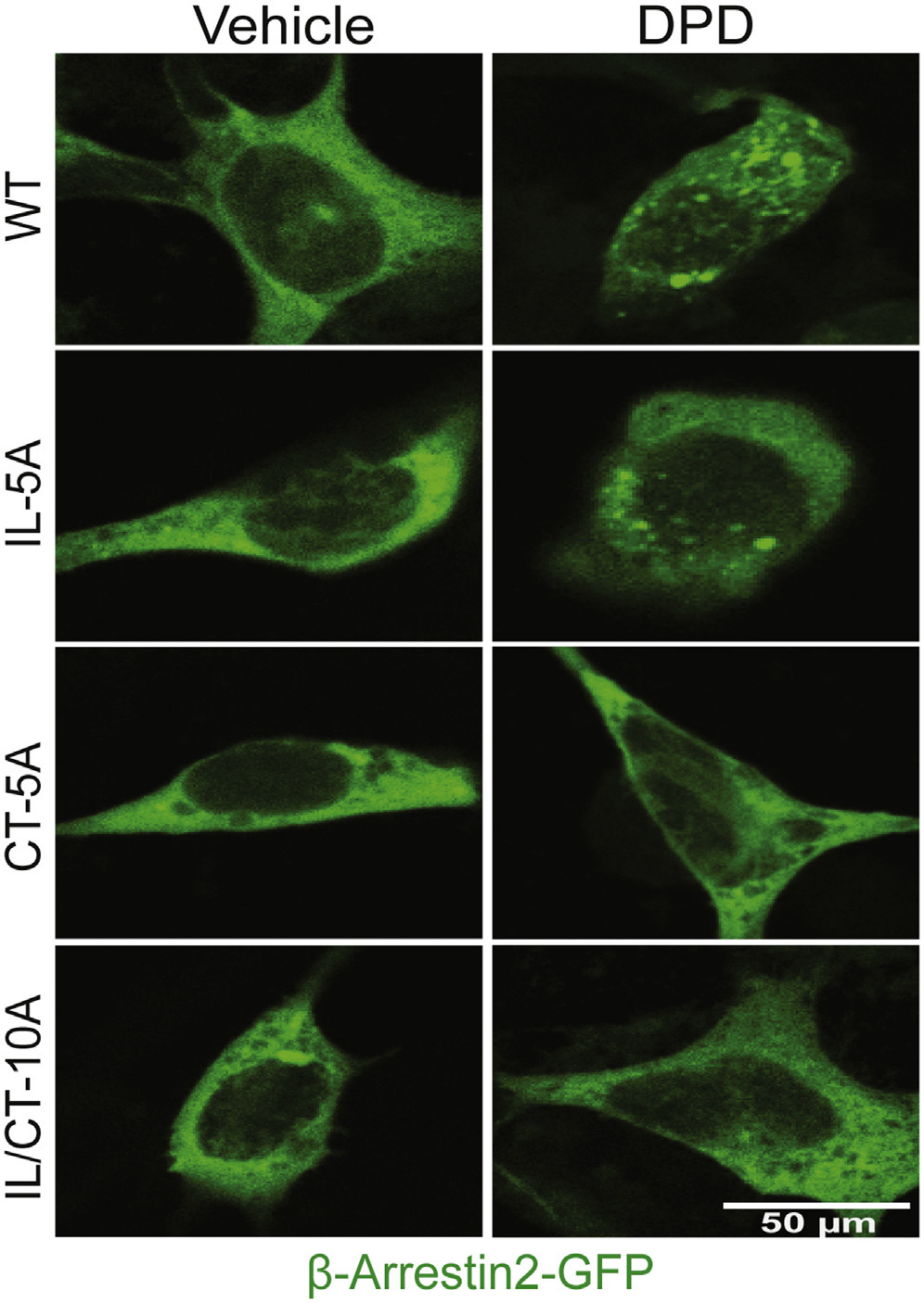
**Qualitative assessment of agonist-promoted β-arrestin2 recruitment by WT and mutant TAS2R14 receptors.** WT and IL-5A receptors exposed to agonist (10 min) displayed a redistribution of β-arrestin2–GFP from the cytosol to the cell membrane and a punctate accumulation pattern at the cell membrane. This appearance was not observed with CT-5A and IL/CT-10A receptors. Shown is a representative of five experiments performed. CT, carboxy-terminal tail; DPD, diphenhydramine; IL, intracellular loop; TAS2R14, bitter taste 2 receptor member 14.

**Figure 5:**
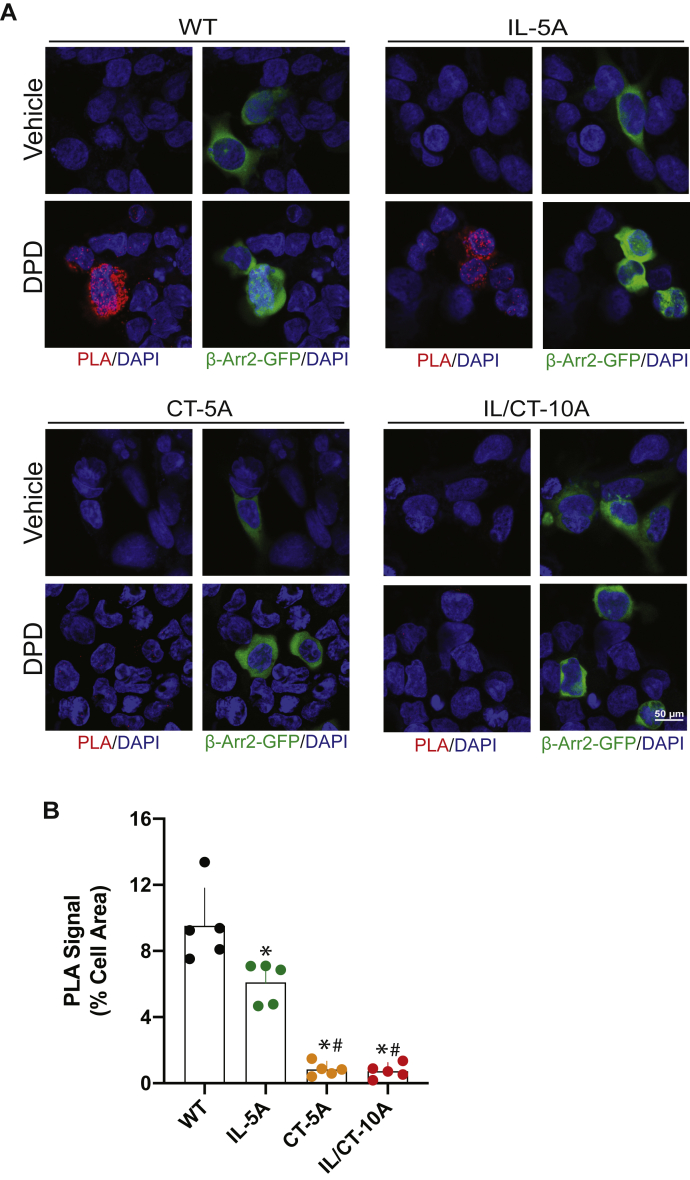
**Proximity ligation assay (PLA) reveals differential β-arrestin2 binding to WT and mutant receptors.***A*, representative experiment showing agonist-promoted β-arrestin2 binding (*red signal*) with the indicated receptors. *B*, mean ± SD, and individual results, from five PLA experiments. ∗*p* < 0.01 *versus* WT; #not significant *versus* 0% change as determined by the one-sample *t* test. DAPI, 4′,6-diamidino-2-phenylindole; DPD, diphenhydramine; IL, intracellular loop.

Agonist-promoted GRK-mediated phosphorylation of most GPCRs results in recruitment of β-arrestins from the cytosol and subsequent binding to the receptor, leading to the uncoupling from the G protein and desensitization. We reasoned that recruitment would be impaired with one or more of the phosphodeficient mutant receptors. Using cells transfected with GFP–β-arrestin2, the translocation of β-arrestins is observed under confocal microscopy as a loss of the intracellular signal and a punctate appearance of GFP–β-arrestin2 at the cell surface (21, 22). As an initial qualitative screen, we assessed whether this characteristic response is altered with any of the mutated receptors. As indicated in Figure 4, WT TAS2R14 displayed the aforementioned phenotype, as we have recently described (22). IL-5A receptors also recruited GFP–β-arrestin2 in an agonist-dependent manner similar to that of WT. However, there was no evidence of redistribution or the appearance of puncta at the cell surface with IL/CT-10A or CT-5A (Fig. 4). To quantitatively assess the binding of β-arrestin2 to the WT and mutant receptors, we utilized a proximity ligation assay (PLA) that we have recently described in detail (see Experimental procedures section) (23). A signal in the red spectra is produced when the two proteins of interest (β-arrestin2 and one of the receptors) are within 30 nm. Figure 5*A* shows a representative PLA with the four receptors, with mean data from multiple experiments found in Figure 5*B*. Negative controls were experiments that were performed lacking the primary antibody to GFP or FLAG, or the PLA probes, which showed no signals (Fig. S4), as well as experiments with cells expressing WT receptor that emitted no signal in the absence of agonist (Fig. 5*A*). With the WT receptor, a robust PLA signal upon exposure of the cells to agonist was readily apparent after a 10-min incubation. At the other extreme, IL/CT-10A, which lacks all potential GRK-mediated phosphorylation sites, showed no agonist-promoted PLA signal (no difference from 0% by the one-sample *t* test; Fig. 5, *A* and *B*), consistent with a lack of β-arrestin2 recruitment with this mutated receptor. Similarly, the CT-5A mutant lacking the five Ser displayed no agonist-promoted PLA signal. For the IL-5A receptor, we found a significant agonist-promoted PLA signal, but on average, it was ∼35% lower than that observed for R14-WT (Fig. 5*B*). This modest decrease observed with IL-5A suggested that the third loop phosphorylation sites may play a role in β-arrestin2 binding and function that is distinct from the rapid uncoupling event.

### Internalization and early endosome colocalization of TS2R14 requires GRK–phosphoacceptor sites in the IL3 and CT

**Figure 6:**
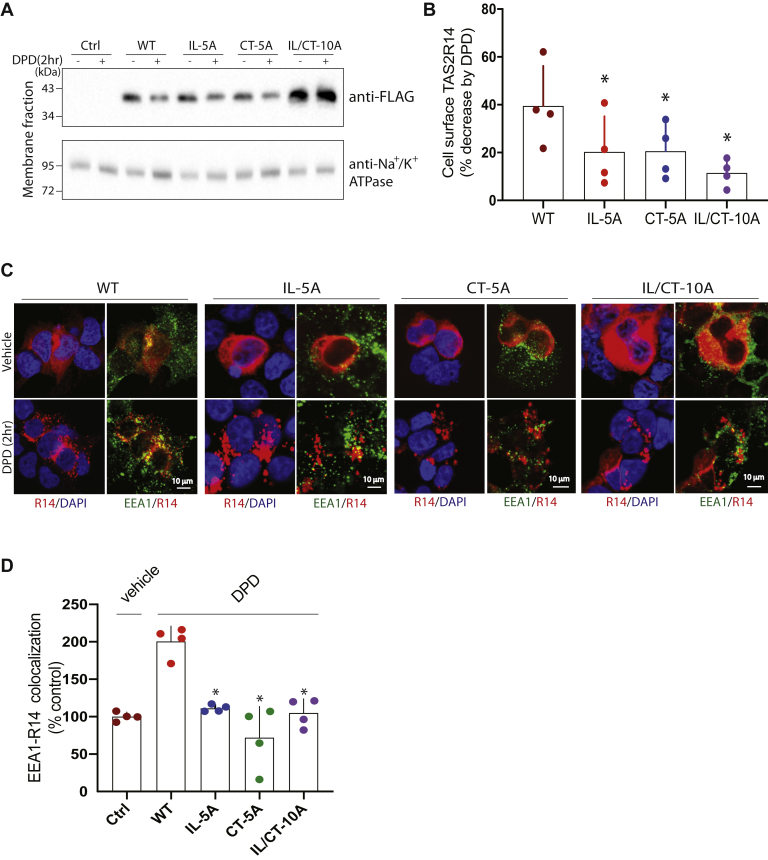
**Agonist-promoted TAS2R14 internalization and sorting to early endosomes is altered by Ser/Thr mutations.***A*, representative experiment showing agonist (DPD)-promoted loss of cell surface receptors as determined by Western blotting of the membrane fraction. *B*, mean ± SD, and individual results, from four cell surface expression experiments. ∗*p* < 0.05 *versus* WT. *C*, agonist-promoted colocalization (*yellow signal*) of WT receptor with the early endosome marker EEA1, which was not observed with any of the indicated mutant TAS2R14 receptors. Shown is a representative experiment. The scale bars are shown in the bottom right-hand panel for each cell line. *B*, mean ± SD, and individual results, from four colocalization experiments. ∗*p* Value not significantly different compared with vehicle control. CT, carboxy-terminal tail; DAPI, 4′,6-diamidino-2-phenylindole; DPD, diphenhydramine; EEA1, early endosome antigen 1; IL, intracellular loop; TAS2R14, bitter taste 2 receptor member 14.

With continuous agonist exposure, many GPCRs (13), including TAS2R14 (22), internalize to the cell interior and begin to undergo endosomal degradation. We have shown that TAS2R14 internalizes and associates with the early endosomal marker early endosome antigen 1 (EEA1) (22) under conditions of prolonged agonist exposure. A time course for receptor–EEA1 colocalization shows that steady state was reached after 2 h of agonist exposure (Fig. S5). Based on the partial loss of β-arrestin2 binding with IL-5A, we assessed receptor internalization and EEA1 colocalization with WT and each of the mutated receptors. Cells were treated in culture plates with the agonist DPD or vehicle at 37 °C for 2 h, cooled to 4 °C, washed, and cell membrane fractions derived. These fractions were probed with the FLAG antibody in immunoblots along with the Na^+^/K^+^ ATPase as a membrane protein marker (Fig. 6, *A* and *B*). WT receptor exhibited a 40% loss of cell surface receptors under these conditions. In contrast, IL-5A, CT-5A, and IL/CT-10A displayed an impairment of internalization, with the latter showing the smallest degree of cell surface loss. We ascertained agonist-promoted colocalization of receptors with EEA1 under the same conditions, using confocal microscopy in permeabilized cells for detection (Fig. 6, *C* and *D*). WT showed agonist-promoted colocalization of receptor with EEA1 (*yellow signal*; Fig. 6*C*). In contrast, experiments with the IL/CT-10A and CT-5A receptors showed very little colocalization. Importantly, the IL-5A receptor also failed to colocalize, indicating a role for these IL3 GRK phosphorylation sites in internalization and trafficking that are distinct from the uncoupling event. The adapter functions of β-arrestin serve to internalize GPCRs and also promote β-arrestin–dependent extracellular signal–regulated kinase 1/2 (ERK1/2) activation. In our hands, nontransfected HEK-293T cells have a high ERK1/2 off-target response to TAS2R agonists, so we were unable to study the receptors in this cell type. However, HASM cells, which endogenously express this receptor, show a TAS2R14-specific ERK1/2 response (24, 25). We thus studied these cells in the absence and presence of an internalization inhibitor (dynasore). As shown in Figure S6, dynasore inhibited agonist-dependent ERK1/2 activation by ∼50%. By inference from studies shown in Figures 5 and 6, we consider that ERK1/2 activation may also require the phosphorylation sites in both the third loop and CT regions of the receptor.

## Discussion

TAS2Rs are GPCRs that were thought to be expressed exclusively on taste cells of the tongue, having evolved to detect toxic bitter substances from plants (26). Recent studies, however, have shown that many of the 25 human TAS2R subtypes are expressed on multiple extragustatory cell types within various organs in the body. Examples include airway smooth muscle, brain, thyroid, pancreas, gastrointestinal tract, white blood cells, and uterus (27, 28). Thus, it is now clear that a previously unrecognized TAS2R-based chemosensory system is present in the body, which may act in a regulatory, compensatory, or pathogenic manner in normal homeostasis or in disease. Based on this distribution and functional outcomes from receptor activation, TAS2Rs may represent potential novel drug targets as well (19). Activation of TAS2R14, which is highly expressed on HASM cells, causes marked relaxation of the cells and dilation of the bronchus, which appears to be of greater magnitude (29) than the only direct bronchodilators (β_2_-adrenergic receptor [β_2_AR] agonists) available for treating airway constriction in asthma and chronic obstructive pulmonary disease. The expression and function of TAS2Rs is not altered in HASM cells derived from asthmatic donor lungs compared with nonasthmatic lungs (30), confirming the availability of the target in the diseased state. TAS2Rs couple to the G_i_ family of G proteins (24) (G_gust_ in taste cells and G_i1,2,3_ in HASM cells). Upon binding to the α-subunit, the released βγ of the heterodimeric G protein activates phospholipase C, leading to diacylglycerol and inositol triphosphate production, the latter inducing Ca^2+^ release from the sarco(endo)plasmic reticulum *via* the inositol triphosphate receptor. In HASM cells, the increase in [Ca^2+^]_i_ in microdomains acts to hyperpolarize the cell membrane and relax the cell (19). Thus, [Ca^2+^]_i_ is a readily measured second messenger representing receptor–G protein coupling.

Here, we show that TAS2R14 indeed undergoes rapid agonist-promoted desensitization of the [Ca^2+^]_i_ response that is dependent on GRK2 phosphorylation. GRK-mediated desensitization has been demonstrated for multiple other GPCRs, typically at Ser/Thr in the cytoplasmic C terminus such as the β_2_AR (4) or the μ-opioid receptor (31), or less frequently in the IL3 such as the α_2A_AR (3) and the M2-muscarinic receptor (32). Uncharacteristically, TAS2R14 has a small IL3 (26 compared with 158 amino acids of the α_2A_AR, where the GRK sites are found) as well as a small cytoplasmic C terminus (30 *versus* 84 amino acids of the β_2_AR, where the GRK sites are found for this receptor). Thus, TAS2R14 is compact, and prior to this study, it was not clear whether a GRK-based mechanism was in play with this receptor with such small intracellular domains. We found that using TAS2R14 peptides that purified activated GRK2 phosphorylates both the intracellular loop and cytoplasmic tail regions *in vitro* and failed to do so with the same peptides having Ala substituted at the Ser/Thr candidate sites. In the *in cellulo* studies, mutation of the five phosphoacceptor sites in the third loop along with the five in the C terminus (IL/CT-10A) ablated desensitization. And, knockdown of GRK2 resulted in a signaling phenotype of WT receptor that was quite similar to IL/CT-10A (compare Fig. 2*A* with Fig. 3*B*). Furthermore, β-arrestin recruitment, and binding, to TAS2R14 was absent with this mutated receptor. Since we have previously shown that agonist-initiated TAS2R desensitization is insensitive to PKC inhibitors (33), the current work strongly implicates a GRK-mediated mechanism of desensitization of this receptor. The TAS2R14 receptor lacking the five Ser in the CT (CT-5A) showed a lack of desensitization and β-arrestin engagement that was identical to the IL/CT-10A mutant, localizing the critical GRK phosphorylation sites, relevant to uncoupling, to this region. For these functional desensitization studies, the third loop mutant IL-5A had a pattern that was not quantitatively different than WT, again consistent with the CT being the relevant site for GRK phosphorylation as it relates to uncoupling. While quantitative measurements of β-arrestin association with IL/CT-10A and CT-5A revealed very low signals that were not different than background, IL-5A showed a significant association with β-arrestin. However, it was nevertheless ∼35% less than WT. This suggested that the third loop β-arrestin interactions, possibly in conjunction with the CT sites, might contribute to β-arrestin–mediated functions other than G protein uncoupling. Receptor internalization and ERK1/2 phosphorylation were considered. Both events are dependent on active β-arrestin conformations, which promote the adapter functions of the protein. We found that in fact IL-5A had impaired internalization despite having a WT CT, confirming a role for β-arrestin binding at the third loop in this process. As expected (because of the complete loss of β-arrestin engagement), IL/CT-10A and CT-5A also had impaired internalization. In addition, while WT receptor showed colocalization with early endosomes as a result of agonist-promoted internalization, none of the mutants displayed this colocalization. Thus, WT internalization requires both the sites in the IL3 and those of the CT, while desensitization requires only the CT sites.

The divergence of β-arrestin functions of GRK-phosphorylated Ser/Thr at different intracellular receptor domains is unusual but not without precedence. For example, there are two clusters of Ser/Thr in the IL3 of the M2-muscarinic receptor (32). Both are phosphorylated by GRK2, but only one specific cluster is required for agonist-promoted desensitization. On the other hand, phosphorylation of either cluster is sufficient for receptor internalization. For the D2-dopamine receptor, eight Ser/Thr in the IL3 are phosphorylated by GRK2/3; their mutation to Ala alters receptor trafficking but not agonist-promoted desensitization (34). Cooperative and hierarchical phosphorylation of intracellular residues by GRKs has also been reported, such as with the δ-opioid receptor (35), adenosine A3 receptor (36), and D1-dopamine receptor (37), the latter involving sites in the third loop and the CT. It may be that many GPCRs in the superfamily have evolved various unique GRK/β-arrestin–initiated mechanisms to optimize receptor regulation for a given receptor. Indeed, there may be no prototypic GRK-based mechanism that is applicable to most receptors. For TAS2R14, we find that the uncoupling function (desensitization) requires GRK sites localized exclusively to the CT. In contrast, agonist-promoted internalization requires GRK phosphorylation sites in the third loop as well as the CT.

We also note that the steady-state maximal level of internalization for TAS2R14 occurs with ∼2 h of agonist exposure. This time to reach maximal internalization is longer than for many GPCRs, such as the β_2_AR (38), where maximal internalization levels occur with <30 min of agonist exposure. Since internalization removes receptors from the cell surface, this mechanism can act as a second, delayed, component of agonist-promoted desensitization of the cellular response. The extent and kinetics of internalization are known to vary among GPCRs (14, 38, 39, 40). This is true for receptors within families, such as the β_2_AR *versus* α_2A_AR in the adrenergic family (38, 39), and even within closely related receptors of a subfamily, such as the β_1_-, β_2_-, and β_3_AR subtypes (14, 38) and α_2B_AR *versus* α_2C_AR (40). These differences may be due to divergent evolutionary pressures for desensitization during normal physiologic conditions or adaptive responses in disease. For TAS2Rs, the relatively slow internalization may represent resistance to desensitization, since these receptors are thought to have developed on taste cells as a warning mechanism to avoid ingestion of toxins from plants (41). Thus, a more protracted period after toxin exposure before this second component of desensitization is fully underway, may be a desirable feature for a bitter taste receptor in this context. Given that internalization requires the adapter functions of activated β-arrestins, we also examined agonist-mediated activation of ERK1/2, which for many GPCRs including TAS2R14 (25), is mediated in part by β-arrestin. We found that inhibition of dynamin, which blocks internalization, also partially inhibited agonist-promoted ERK1/2 activation in HASM cells. Since receptors with mutations of GRK phosphorylation sites in the IL3 or the CT failed to internalize, we also propose that GRK phosphorylation in both regions may also be necessary for TAS2R14-mediated ERK1/2 activation.

Our results are readily applicable to the consideration of utilizing TAS2R14 agonists for the treatment of obstructive airways owing to HASM contraction. Currently, β-agonists acting at β_2_AR on HASM (*via* G_αs_ coupling to cAMP production) are the only direct bronchodilators available. The clinical need for a different class of direct bronchodilators to treat obstructive lung diseases such as asthma has been promoted for a number of years (42, 43). β-agonist use in asthma has been associated with multiple adverse effects, including tachyphylaxis (44, 45), increased responsiveness to constrictive stimuli (46, 47), loss of the protective effect of β-agonist to constrictive stimuli (48, 49), increased exacerbations (50, 51), and death (50, 52, 53). The use of a direct bronchodilator that has a different mechanism of action, such as agonists to TAS2R14, might circumvent some of these limitations of β-agonists. Using an unbiased approach to search for new targets, we probed HASM for transcripts representing all known or suspected GPCRs (54). The unexpected finding of TAS2R expression on these extragustatory cells (at levels equivalent to β_2_AR) prompted studies to assess their function in HASM, as potential therapeutic targets with an alternative mechanism of action (19, 29, 30). These studies indicated that agonists promote significant relaxation and markedly dilate bronchi in a [Ca^2+^]_i_-dependent manner. Consistent with their different modes of action, studies showed that submaximal doses of β-agonist and TAS2R agonist bronchodilate in an additive fashion, suggesting that both classes of agonists might be used concomitantly to treat airway obstruction (19).

In conclusion, we show that TAS2R14 undergoes rapid agonist-promoted desensitization, amounting to ∼50% loss of function. The molecular determinants of this desensitization were ascertained using *in vitro* and cell-based studies with mutated sequences of the IL3 and cytoplasmic CT. GRK2 was implicated as the kinase responsible for phosphorylation based on the *in vitro* studies of peptide sequences, GRK2 knockdown functional studies in endogenously expressing cells, and mutagenesis of the receptor. This phosphorylation leads to recruitment and binding of β-arrestin2 to the receptor resulting in uncoupling from G_αi_. Ser residues in the CT were found to be required for GRK-mediated β-arrestin2 binding and functional desensitization, whereas Ser/Thr in the IL3 as well as the CT were required for receptor internalization and entry into the degradation pathway.

## Experimental procedures

### Expression constructs, mutagenesis, cell culture, and transfection

The WT N-terminal FLAG-tagged TAS2R14 construct based on the vector pCDNA3 (55) was utilized to generate three mutant TAS2R14 receptors (Fig. 1*A*) using site-directed mutagenesis methods as previously described (3). The mutant denoted IL-5A consisted of substitutions of all Ser/Thr in the IL3 with Ala (residues 215, 219, 223, 224, and 232). CT-5A consisted of substitution of the Ser residues to Ala in the cytoplasmic tail (residues 291, 293, 308, 316, and 317). The IL/CT-10A mutant consists of all 10 substitutions in both domains. Plasmids were sequenced to confirm the mutations and to exclude off-target mutations. HEK-293T and D9 human telomerase reverse transcriptase–immortalized HASM cells (24) were cultured at 37 °C and 5% CO_2_ in Dulbecco's modified Eagle's medium containing 10% fetal bovine serum, 100 units/ml of penicillin, and 100 μg/ml of streptomycin. Cells were seeded in 60 mm Petri dishes at a density of 0.5 × 10^6^ cells/dish. Following overnight growth, cells were transfected to achieve transient expression using polyethylenimine (Polysciences) or Lipofectamine 2000 (Thermo Fisher Scientific) as described (24, 55). siCtrl and siGRK2 constructs were from Dharmacon (catalog nos. D00121-01 and M-004325-02). For the microscopy-based desensitization studies, cells were seeded at 40,000 cells/well the night before.

### GST fusion proteins

GST fused TAS2R14 IL3 and cytoplasmic tail complementary DNA was generated using TAS2R14-WT and TAS2R14 to 10A as templates. GST-TAS2R14-IL3–specific primers were 5′cgcggatccatgatcttctccatgtgg3′ (forward) and 5′ccgctcgagtttaactcctctgtgggc3′ (reverse). GST-TAS2R14-CT–specific primers were 5′cgcggatccatgaacaagaagctgagac3′ (forward) and 5′ccgctcgagagatgattctctaaattct3′ (reverse). The PCR products were cloned into the prokaryotic expression vector pGEX-4T1 (GE Healthcare Bio-Sciences) at the *Bam*HI-*Xho*I sites. Transformed *Escherichia coli*, strain BL21 (DE3), was treated with isopropyl β-d-1-thiogalactopyranoside to induce protein expression. Protein purification was performed using glutathione Sepharose 4B (GE Healthcare Bio-Sciences) as previously described (56).

### *In vitro* phosphorylation

Assays were performed with 10 μCi of γ-^32^P-ATP (PerkinElmer) and 10 μM of unlabeled ATP in 30 μl of buffer containing 20 mM Tris–Cl (pH 8.0), 4 mM MgCl_2_, active GRK2 recombinant protein (0.2 μM; Sigma), and the GST-tagged fusion proteins as substrates. After incubation at 30 °C for 30 min, the reactions were stopped by adding protein loading buffer and the proteins electrophoresed through 12% SDS-PAGE gels. The relative amounts of incorporated radioactivity were determined by autoradiography and quantitated by Image J (National Institutes of Health). Negative controls consisted of the reaction without GRK and the reaction in the presence of activated protein kinase B (where the peptides have no predicted phosphorylation sites for this kinase).

### Agonist-promoted desensitization

Transfected cells were transferred onto eight-well chamber slides and 48 h later loaded with Fluo-4 followed by treatment with 200 μM of the agonist DPD for the indicated time. Images were acquired using the EVOS Cell Imaging System (Thermo Fisher Scientific) with excitation at 488 nm and a 515 to 540 nm emission filter. Desensitization was defined as the decrease in the [Ca^2+^]_i_ signal after the peak response (which occurs at 10 s after agonist exposure) to 50 s, and the slope of the decrease was determined for each experiment and used for statistical comparisons between mutant receptors.

### PLA and β-arrestin recruitment assay

To determine the redistribution and binding of β-arrestin to TAS2R14, HEK-293T cells were transfected on coverslips (10,000 cells per coverslip) with constructs expressing β-arrestin2–GFP (0.5 μg) and WT-FLAG or the aforementioned mutant receptors (0.5 μg), and a PLA was performed as recently described (22). Cells were exposed to vehicle or 500 μM DPD for 10 min at 37 °C and fixed with 4% paraformaldehyde. Cells were imaged by fluorescence confocal microscopy (40× magnification, 3× zoom) with 591-nm excitation and 614-nm emission wavelengths to acquire the red spectra (the PLA signal) and 489-nm excitation and 510-nm emission wavelengths to visualize the receptor–GFP signal. For each experiment, five to seven images were acquired per condition per transfected construct. Transfected cells (indicated by the presence of the GFP signal) were analyzed by ImageJ to quantitate the red PLA puncta, with results presented as the percentage of the area of the cells occupied by puncta. These quantitative results were complemented with qualitative studies of agonist-promoted β-arrestin–GFP recruitment from the cytosol to the cell membrane, where β-arrestin–GFP appears as punctate accumulations at the membrane (21). Briefly, HEK-293T cells were transiently transfected on coverslips with constructs encoding β-arrestin2–GFP and the TAS2R14 constructs indicated previously. Cells were treated with vehicle or 200 μM DPD for 10 min and then fixed with 4% paraformaldehyde. The effect of agonist exposure was visualized by confocal microscopy (40× magnification, 3× zoom).

### Agonist-promoted TAS2R14 internalization and early endosome colocalization

Agonist-promoted receptor internalization was defined as the loss of cell surface receptors after 2 h of incubation with 500 μM DPD at 37 °C compared with vehicle control. The cell membrane fraction was obtained using Mem-PER plus (catalog no. 89842; Thermo Scientific) as previously described (55). Briefly, transfected HEK-293T cells expressing WT or mutant receptors were washed, scraped from the plate, pelleted by centrifugation, and solubilized in the Mem-PER buffer with constant rotation 20 min at 4 °C. After centrifugation for 15 min at 16,000*g*, the pellet representing the cell membrane fraction was solubilized and probed by immunoblotting for FLAG (antibody titer 1:1000) and the Na^+^/K^+^ ATPase (exclusively expressed on the cell membrane; titer 1:1000) as a loading control. Studies to colocalize receptor with early endosomes were performed with HEK-293T cells transfected on coverslips with constructs (1.0 μg) to express TAS2R14WT-FLAG and the aforementioned mutant receptors. About 48 h after the transfection, cells were exposed to vehicle or 500 μM DPD for 2 h at 37 °C and fixed with 4% paraformaldehyde. Cells were incubated with primary antibodies (EEA1, 1:100 and FLAG, 1:100) at 4 °C overnight, and secondary antibodies (Alexa Fluor 488 and Alexa Fluor 594) at 1:1000 at room temperature for 45 min. Cells were imaged by fluorescence confocal microscopy (40× magnification, 2× zoom) with 591-nm excitation and 614-nm emission wavelengths to acquire the red spectra (FLAG) and 493-nm excitation and 519-nm emission wavelengths to acquire the green spectra (EEA1). Antibodies for these experiments and others described previously were from the following sources with the indicated catalog numbers: FLAG (F3165; Sigma), Na^+^/K^+^ ATPase (3010; Cell Signaling), Alexa Fluor 488 and 594 (A32766 and A32740; Invitrogen), EEA1 (3288; Cell Signaling), and GFP (8344; Santa Cruz).

### Statistical analysis

Bands from Western blots were quantitated by ImageJ, and the pixel density was compared by paired or unpaired *t* tests. The PLA signals were compared by ANOVA followed by paired *t* tests between R14-WT and the mutant receptors. The PLA signals and ^32^P signals from the *in vitro* phosphorylation studies for the mutants were also compared with 0 (*i.e.*, no signal) using the one-sample *t* test (*p* < 0.01 considered significant). For desensitization experiments, the [Ca^2+^]_i_ data from 10 to 50 s were fit by linear regression. For each individual experiment, the slopes were calculated based on the best fit regression and were compared by *t* tests. Unless otherwise stated, *p* values <0.05 were considered significant. Bar graphs are presented as mean ± SD along with the results from the individual experiments.

## Data availability

All data described in the article are present in the main text, figures, and the supporting figures.

## Conflict of interest

The authors declare that they have no conflicts of interest with the contents of this article.
