# Supplementary material for: The short third intracellular loop and cytoplasmic tail of bitter taste receptors provide functionally relevant GRK phosphorylation sites in TAS2R14
Source: J Biol Chem. 2021 Jan 16;296:100216. doi: 10.1074/jbc.RA120.016056 (PMC7949105; doi:10.1074/jbc.RA120.016056)
Supplement: Figures S1 to S5 [file mmc1.pdf]

The short third intracellular loop and cytoplasmic tail of bitter taste receptors provide functionally relevant GRK phosphorylation sites in TAS2R14

Donghwa Kim[1], Maria Castaño[1], Lauren K. Lujan[1], Jung A. Woo[2], Stephen B. Liggett[3]

[1]Department of Medicine, University of South Florida Morsani College of Medicine, Tampa, FL, USA; [2]Department of Molecular Pharmacology and Physiology, University of South Florida Morsani College of Medicine, Tampa, FL, USA; [3]Departments of Medicine and Molecular Pharmacology and Physiology, University of South Florida Morsani College of Medicine, Tampa, FL, USA

*Corresponding author: Stephen B. Liggett: 12901 Bruce B. Downs Blvd., MDC 2, Tampa, FL, 33635. Email: sliggett@usf.edu; Tel: (813) 974-7715.

Running Title: TAS2R14 regulation

Keywords: TAS2R, desensitization, G protein coupled receptors, G protein coupled receptor kinases, β-arrestin

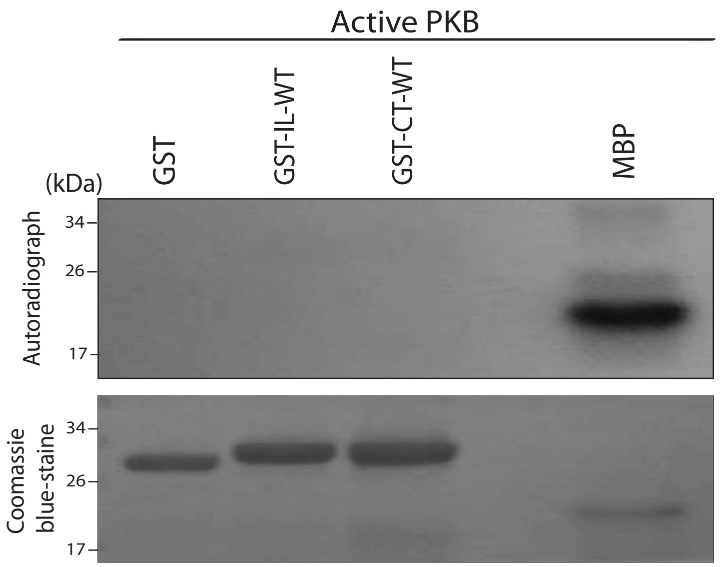

Supplemental Figure S1. Negative control for the in vitro phosphorylation assays. Activated PKB fails to phosphorylate the WT TAS2R14-GST fusion proteins derived from the IL or CT of the receptor. Purified Myelin Basic Protein (MBP), a known substrate for PKB, is phorphorylated as indicated. Representative experiment from 3 performed.

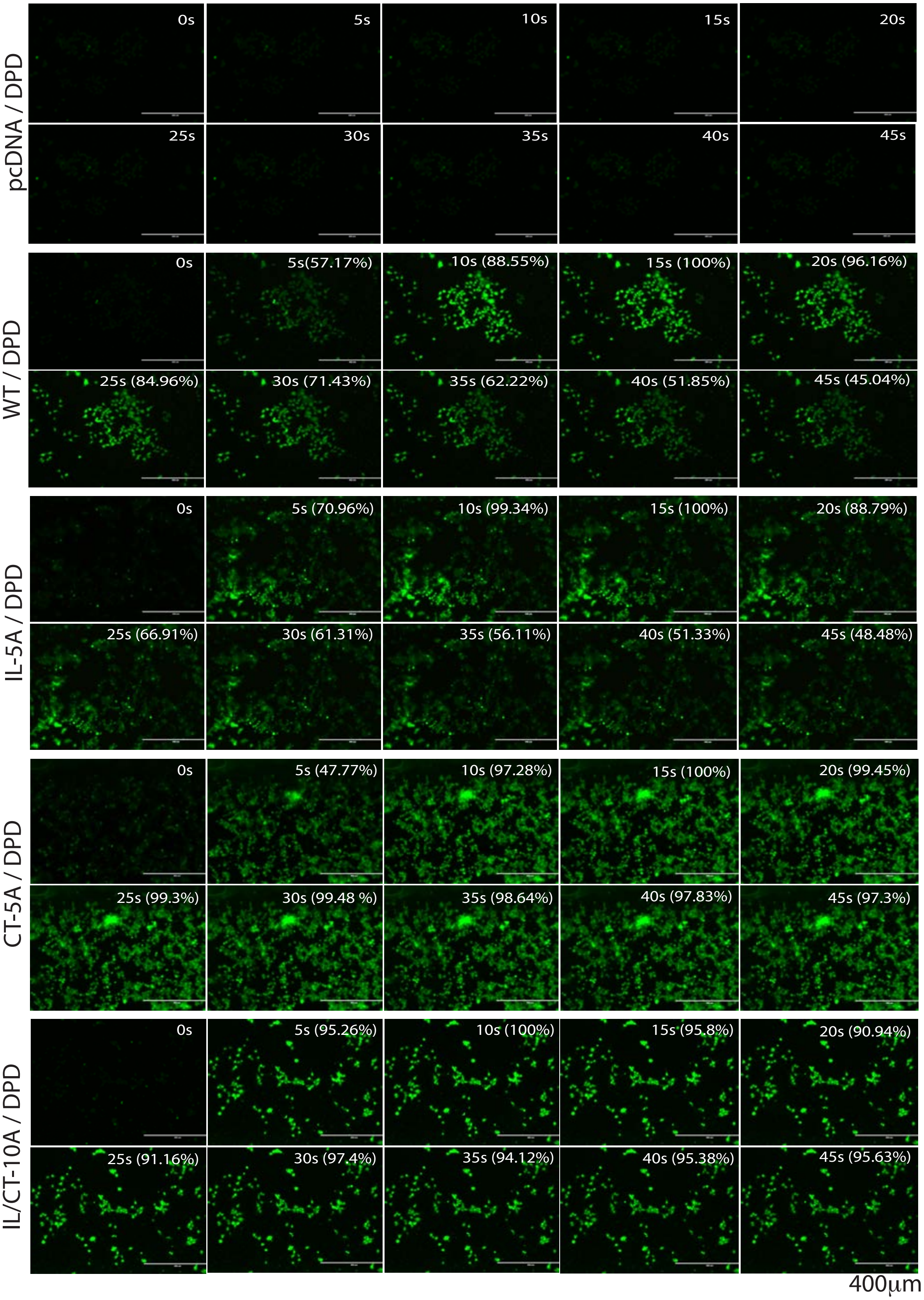

Supplemental Figure S2. Agonist-promoted desensitization of the $[Ca^{2+}]_i$ response from TAS2R14 activation. HEK-293T cells were transfected with pcDNA3 (control), WT, or the indicated mutated TAS2R14 constructs. Cells were loaded with Fluo-4, exposed to 500 μM DPD, and fluorescence imaged by confocal microscopy at the indicated time points as described in Materials and Methods. In parenthesis the amplitude of the signal from the field is shown as a percentage of the maximal signal (usually observed at 10 or 15 sec). Shown is a representative experiment of 5 performed. See Fig. 2 for mean results.

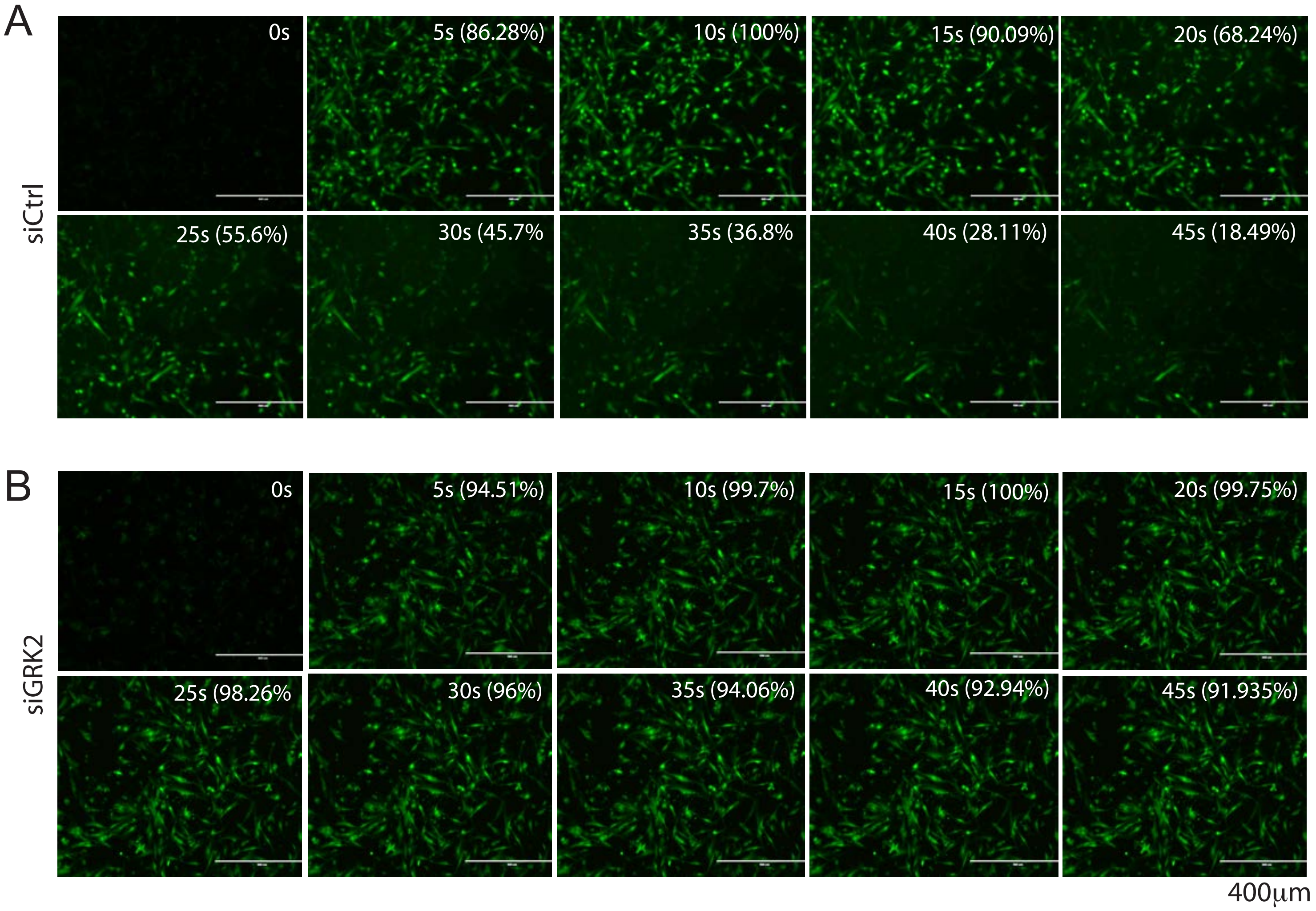

Supplemental Figure S3. **Agonist-promoted desensitization of the $[Ca^{2+}]_i$ response of TAS2R14 activation under GRK2 knockdown conditions.** HASM cells were loaded with Fluo-4, exposed to 500 μM DPD, and fluorescence imaged by confocal microscopy at the indicated time points as described in Materials and Methods. In parenthesis the amplitude of the signal from the field is shown as a percentage of the maximal signal (usually observed at 10 or 15 sec). Shown is a representative experiment of 5 performed. See Fig. 3 for mean results. Shown is a representative experiment of 5 performed in HASM cells.

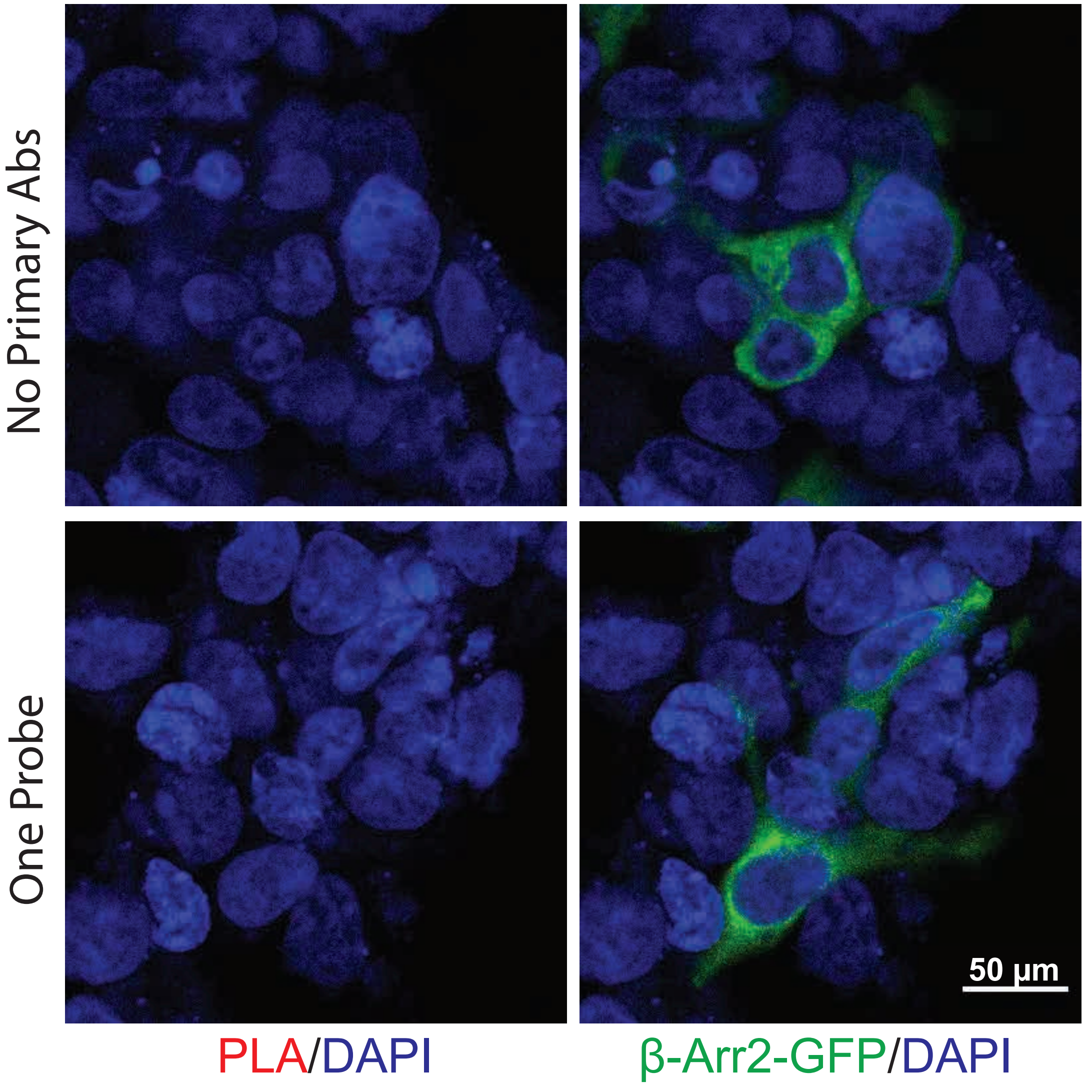

Supplemental Figure S4. Negative controls for the PLA assay. HEK-293T cells transfected with WT TAS2R14 and β-arrestin2-GFP were treated with 500 μM DPD and the assay was performed without the two primary antibodies (top two panels) or without one of the the PLA probes (bottom two panels). A PLA signal (red puncta) was not observed under either condition. The green signal represents fluorescense of β-arrestin2-GFP. Results are representative of 4 experiments. Positive PLA signals are shown in Fig. 5.

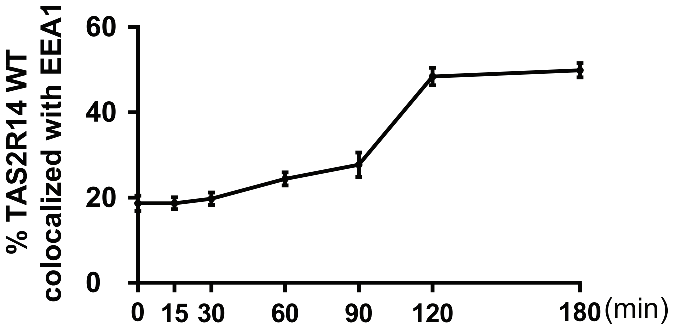

Supplemental Figure S5. Time course of agonist-promoted TAS2R14 colocalization with the early endosome marker EEA1. Transfected HEK-293T cells were exposed to the agonist DPD (500 uM) for the indicated times and colocalization was ascertained by confocal microscopy as described in Materials and Methods. Results are mean +/- SE from 3 experiments.

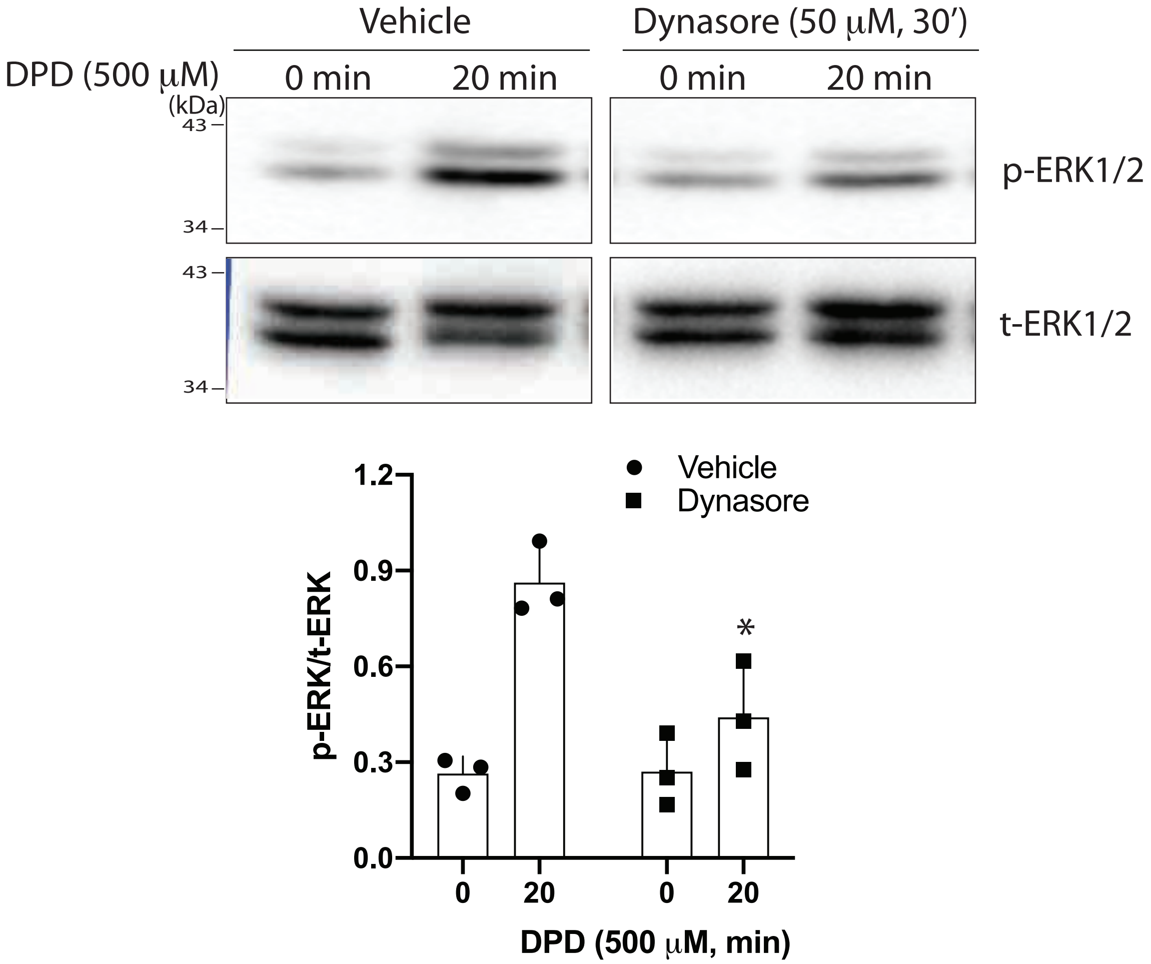

Supplemental Figure S5. An inhibitor of internalization partially blocks TAS2R14-mediated activation of ERK1/2. A) representative experiment of 3 performed. B) Results from 3 experiments. *, $P<0.05$ vs DPD stimulated vehicle treated cells
